# Combination therapy of KRAS G12V mRNA vaccine and pembrolizumab: clinical benefit in patients with advanced solid tumors

**DOI:** 10.1038/s41422-024-00990-9

**Published:** 2024-06-24

**Authors:** Xinjing Wang, Wei Wang, Siyi Zou, Zhiwei Xu, Dan Cao, Shuai Zhang, Minzhi Wei, Qian Zhan, Chenlei Wen, Fanlu Li, Hao Chen, Da Fu, Lingxi Jiang, Ming Zhao, Baiyong Shen

**Affiliations:** 1 Department of General Surgery, Pancreatic Disease Center, Ruijin Hospital, Shanghai Jiao Tong University School of Medicine, Shanghai, China; 2 Research Institute of Pancreatic Diseases, Shanghai Key Laboratory of Translational Research for Pancreatic Neoplasms, Shanghai Jiao Tong University School of Medicine, Shanghai, China; 3 State Key Laboratory of Oncogenes and Related Genes, Institute of Translational Medicine, Shanghai Jiao Tong University, Shanghai, China; 4 Shanghai Xinpu BioTechnology Company Limited, Shanghai, China; 5 Hongene Biotech Corporation, Shanghai, China

**Keywords:** Cancer immunotherapy, Targeted therapies

Dear Editor,

mRNA-based therapeutics have gained the public’s attention in the post-COVID era. The application of mRNA vaccines is not limited to infectious diseases but extends to broader areas, such as cancers and rare genetic disorders.^1–3^ This first-in-human study demonstrated the safety and preliminary efficacy of mRNA vaccines targeting multiple neoantigens in 13 patients with advanced melanoma.^4^ The vaccines significantly reduced recurrent metastatic events, improving progression-free survival. Steven Rosenberg’s group later reported that mRNA vaccines could generate mutation-specific T-cell responses against predicted neoepitopes in 4 patients with metastatic gastrointestinal cancer.^5^ However, no objective clinical responses were observed. Therefore, whether the application of mRNA vaccines can be expanded to cold tumors such as gastrointestinal tumors remains to be addressed. Recently, the efficacy of personalized mRNA vaccines targeting multiple neoantigens in combination with programmed death 1 ligand (PD-L1) inhibitors and mFOLFIRINOX (a modified version of a four-drug chemotherapy regimen) in delaying recurrence in pancreatic cancer patients after surgical resection was reported.^6^ The median recurrence-free survival was not yet reached for vaccine responders (50% of the patients enrolled), compared with an average of 13.4 months for nonresponders. Similarly, personalized mRNA vaccines with up to 34 neoantigens plus the PD-1 inhibitor pembrolizumab slowed recurrence in postsurgical patients with melanoma in the phase IIb KEYNOTE-942 trial.^7^ These early trials suggested that utilizing personalized vaccines to cover multiple neoantigens in the postsurgical setting with a low tumor load is a practical strategy for mRNA therapeutics. Whether a tumor vaccine can be used in late-stage cancer patients with a high tumor load is unknown.

Few studies have reported the efficacy of mRNA vaccines in late-stage cancer patients. This is because late-stage patients usually have a narrow treatment window, while personalized vaccines have a long manufacturing time (8‒12 weeks) from sequencing to bioinformatic prediction and manufacturing. In this case, an off-the-shelf vaccine with a fixed neoantigen could be helpful for late-stage patients. However, such a vaccine requires the neoantigen to be derived from a common mutation in the population. More importantly, the actual presentation of the mutant peptide by human leukocyte antigen (HLA) should be experimentally validated rather than only relying on a computational prediction algorithm. All these key points have made it challenging to discover a shared neoantigen candidate for off-the-shelf tumor vaccines. No vaccine-based strategies targeting a single neoantigen have been reported. Unpublished data analysing all reported immune-responsive neoantigens suggested an extremely low percentage of neoantigen overlap among individuals (< 0.01%, data not shown). As a result, finding a good neoantigen candidate is critical for generating off-the-shelf tumor vaccines for end-stage patients.

KRAS is associated with highly fatal cancers such as pancreatic ductal adenocarcinoma (PDAC) and non-small-cell lung cancer (NSCLC). Several KRAS mutants are presented by human HLAs.^8^ Among multiple peptide‒MHC pairs, KRAS G12V‒HLA-A*11:01 has the strongest binding affinity, the highest success rate of inducing a T-cell response, and the most significant cytotoxic effect on tumor cells.^8^ Using mass spectrometry, we validated the expression and presentation of the KRAS G12V neoantigen delivered through mRNA and its binding to HLA-A*11:01 (Supplementary information, Fig. S1 and Data S1). Wang et al. reported that HLA-A*11:01 transgenic mice exhibited robust T-cell receptor (TCR) reactivity towards the mutated KRAS variants G12V and G12D.^9^ Using this animal model, we demonstrated that the KRAS G12V mRNA vaccine could induce in vivo expansion (Supplementary information, Fig. S2a, b) and activation (Supplementary information, Fig. S2c, d) of G12V-specific T cells and subsequently slow the growth of KRAS G12V-bearing tumors (Supplementary information, Fig. S2e, f). These preclinical results confirm that the KRAS G12V mRNA vaccine can induce antigen-specific cytotoxic T lymphocytes (CTLs).

Here, we report that KRAS G12V is a good neoantigen candidate for people with the HLA-A*11:01 type, and combined therapy with a single neoantigen mRNA vaccine targeting KRAS G12V and the PD-1 inhibitor pembrolizumab induced tumor shrinkage in two patients with end-stage cancer. The immune checkpoint inhibitor pembrolizumab is used here to enhance the function of vaccine-specific tumor-infiltrating lymphocytes (TILs), lower the T-cell activation threshold, and reduce TIL exhaustion. TILs highly express PD-1 and have low cytokine production.^10^ PD-1 blockade leads to improved dendritic cell maturation and consequently higher tumor-specific CD8^+^ T-cell proliferation by enhancing CD154 expression and cytokine secretion by CD4^+^ TILs.^10^ The institutional review board of the Ethics Committee of Ruijin Hospital, Shanghai Jiao Tong University School of Medicine, approved the study. For both patients, whole-exon sequencing and transcriptome sequencing of tumor tissues confirmed the presence of the KRAS c.35 G > T (p.G12V) mutation and HLA-A*11:01. The detected somatic mutations (Supplementary information, Tables S1, S2), PD-L1 expression (Supplementary information, Table S3), complete HLA typing (Supplementary information, Table S4), and predicted neoantigens (Supplementary information, Tables S5, S6) are described in the Supplementary Tables.

Patient-001, an 86-year-old female, presented to the clinic with biliary obstruction and was diagnosed with locally advanced pancreatic head cancer. Considering her age and physical condition (Eastern Cooperative Oncology Group (ECOG) score of 3), surgery and chemotherapy were not pursued. The tumor progressed, and biliary obstruction developed again after biliary stent implantation by endoscopic retrograde cholangiopancreatography (ERCP). The patient chose combined therapy consisting of an mRNA vaccine and pembrolizumab because her disease had progressed (Fig. 1a). The patient experienced manageable adverse effects during treatment, including fever following vaccination. The tumor lesion on the head of the pancreas regressed at the first follow-up after three cycles of treatment (Fig. 1b), and a partial response was observed according to the Response Evaluation Criteria in Solid Tumors (RECIST) 1.1.

**Fig. 1:**
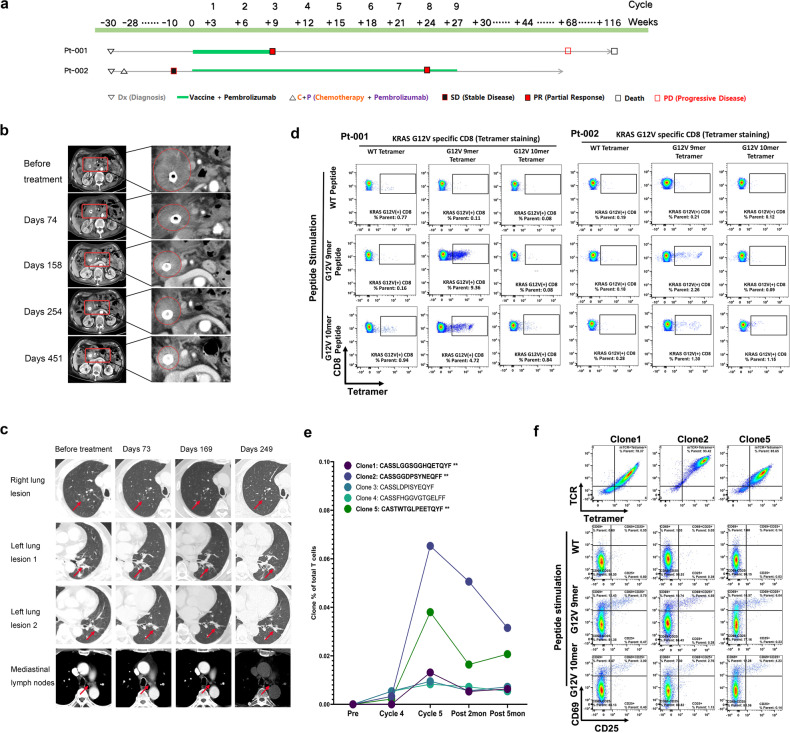
Clinical benefit and specific T cell detection of mRNA vaccine targeting KRAS G12V mutation (HLA-A*11:01 restricted) in advanced solid tumors. **a** Scheme of clinical treatments for patient-001 and patient-002. **b** CT comparison of the tumor lesion of patient-001 before and after the vaccine therapy. **c** CT comparison of multiple tumor lesions of patient-002 before and after vaccination. **d** KRAS G12V-specific CD8^+^ T cells in patient-001 and patient-002 post-vaccination PBMCs. 9 mer (VVGAVGVGK) and 10 mer (VVVGAVGVGK), two short peptides bioinformatically predicted and experimentally proved to be presented by HLA-A*11:01, were added to the culture with PBMCs post-vaccination. On day 10, cells were collected and stained with PE-HLA-A*11:01 tetramer conjugated with KRAS G12V 9 mer, 10 mer, or WT to detect KRAS G12V-specific T cells using flow cytometry. **e** TCR bulk sequencing showed certain T cell clone expansion before and after vaccine. **f** Functional assay validated that T cell clones found in patient-002’s PBMCs (clones 1, 2, and 5) were KRAS G12V specific.

Patient-002, a 69-year-old male, presented pulmonary nodules during an annual physical examination. CT scan and positron emission tomography CT (PETCT) confirmed the presence of multiple hypermetabolic nodules in both lungs and in mediastinal lymph nodes, leading to a pathological diagnosis of advanced NSCLC via CT-guided puncture biopsy. The patient lost the chance for surgery and underwent six cycles of combination therapy consisting of 200 mg/day pembrolizumab and chemotherapy (nab-paclitaxel 400 mg/day 1 + nedaplatin 70 mg/day 1 and day 2) every 3 weeks. Significant regression of the tumor lesion was observed at the first follow-up after three treatment cycles. Later cycles resulted in therapeutic resistance and severe adverse effects, particularly neurotoxicity manifested as limb numbness and fatigue. The patient thus discontinued chemotherapy and chose combination therapy consisting of the mRNA vaccine and pembrolizumab for nine cycles (Fig. 1a). The patient also had no adverse effects other than the expected manageable fever or injection site pain. He developed a partial response according to RECIST 1.1, with the right lung lesion almost cleared, and the left lung lesion and mediastinal lymph node regressed to some extent (Fig. 1c).

We next investigated the immune response related to the combination therapy. We detected HLA-A*11:01-restricted KRAS G12V-specific CD8^+^ T cells in both patients’ postvaccination peripheral blood after KRAS G12V peptide antigen (Fig. 1d) or mRNA-encoded antigen stimulation ex vivo (Supplementary information Fig. S3). The KRAS-specific immune response of patient-002 was de novo (data not shown). Whether the immune response of patient-001 was de novo or pre-existing could not be determined due to the lack of enough clinical material. The intracellular staining data confirmed that the antigen-specific T cells were polyfunctional CTLs and released IFN-γ and TNF-α upon antigen stimulation (Supplementary information Fig. S4). TCR repertoire analyses using TCR bulk sequencing of patient-002’s peripheral blood mononuclear cells (PBMCs) revealed the expansion of several T-cell clones (clones 1, 2, and 5 of Fig. 1e). To clarify whether these clones were KRAS G12V specific, we further stimulated postvaccination PBMCs with the KRAS G12V peptide for 10 days, used FACS to sort HLA-A*11:01‒KRAS G12V tetramer-positive T cells for single-cell TCR sequencing, and found that the expansion of clones 1, 2, and 5 was similar to what we observed on bulk TCR-seq (data not shown). We then transfected the full-length paired TCR-αβ chains back into Jurkat cells. We found that the engineered Jurkat cells were activated once cocultured with PANC-1 cells (a pancreatic cancer cell line expressing HLA-A*11:01) loaded with KRAS G12V but not with KRAS WT or G12D peptide (Fig. 1f; Supplementary information Fig. S5). These results demonstrate that the TCRs in these three clones specifically recognized the KRAS G12V neoepitope.

Besides the clinical benefits, our study highlights the potential of this innovative approach to revolutionize immune therapy in the future. Unlike previous studies reporting mRNA vaccine efficacy in postsurgical patients with low tumor loads,^6,7^ our research focused on patients with advanced solid tumors who missed the window for surgery. An mRNA tumor vaccine targeting a single neoantigen could become a ready-to-use drug, providing clinical feasibility for patients with limited treatment windows. An ideal and appropriate single neoantigen should be derived from a driver mutation and matched with high-frequency HLA subtypes in the population that could strongly present the neoantigen. We understand that such neoantigen–HLA pairs are rare, and KRAS G12V and HLA-A*11:01 are one such pair. Among multiple high-frequency HLA-I alleles, HLA-A*11:01 presents the most diversified KRAS mutants, and KRAS G12V has the strongest binding affinity and stability with HLA-A*11:01.^8,11^ More importantly, KRAS is one of the most commonly mutated driver oncogenes for multiple malignant tumors, such as PDAC.^12^ and NSCLC.^13^ As the HLA-A*11:01 subtype is prevalent in 20%‒60% of the Asian population,^14,15^ the fixed neoantigen vaccine could target ~15% of patients with PDAC, ~5% of patients with colon cancer, and 2.5% of patients with lung cancer.

Finally, this study reported the efficacy of the combination therapy of an mRNA vaccine and checkpoint inhibitor in two patients. Clinical trials demonstrating the benefit of mRNA vaccine monotherapy and reporting the benefit of such immune therapy in a broader population are necessary. Although personalized therapies against tumor neoantigens are an exciting prospect for cancer immune therapy, they are still in the early stages of development. As more neoantigen–HLA pairs are validated, a new perspective on cancer treatment may emerge. Assigning patients to the right treatment based on pathological features and genomic signatures of tumors holds promise.

### Supplementary information

Supplementary Figure 1

Supplementary Figure 2

Supplementary Figure 3

Supplementary Figure 4

Supplementary Figure 5

Supplementary Data S1

Supplementary Table 1

Supplementary Table 2

Supplementary Table 3

Supplementary Table 4

Supplementary Table 5

Supplementary Table 6

## Data Availability

Our transcriptome data, whole exome data, and TCR repertoire diversity data have been deposited in the National Omics Data Encyclopedia (NODE) database, and can be accessed with the accession code OEP004748.
