# Supplementary material for: Combination therapy of KRAS G12V mRNA vaccine and pembrolizumab: clinical benefit in patients with advanced solid tumors
Source: Cell Res. 2024 Jun 24;34(9):661–4. doi: 10.1038/s41422-024-00990-9 (PMC11369195; doi:10.1038/s41422-024-00990-9)
Supplement: Supplementary file 1 — Supplementary Figure 1 [file 41422_2024_990_MOESM1_ESM.pdf]

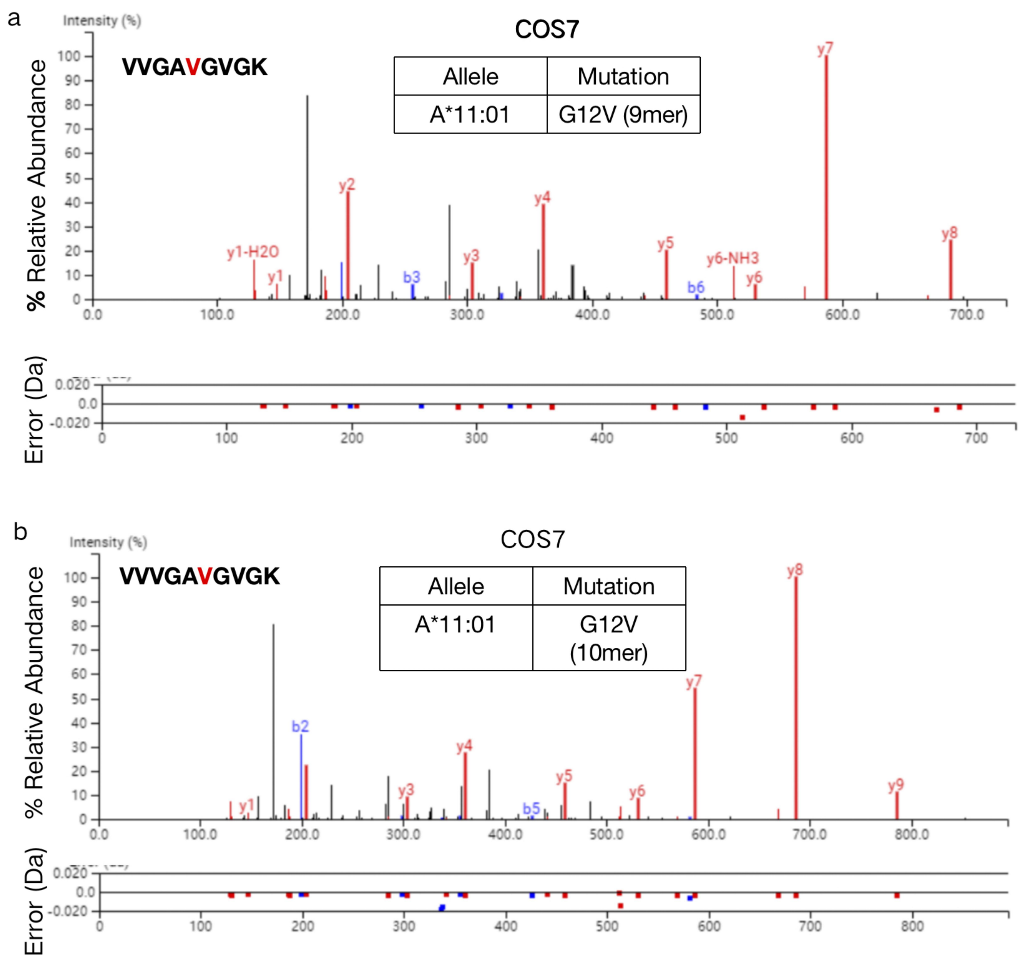

Figure S1. Targeted mass spectrometry of KRAS G12V on HLA-A*11:01. Secondary mass spectrum obtained from LC/MS showed that HLA-A*11:01 was able to present 9 mer peptide (VVGAVGVGK) (a) and 10mer peptide (VVVGAVGVGK) (b) on the cell surface.
