# Supplementary material for: Combination therapy of KRAS G12V mRNA vaccine and pembrolizumab: clinical benefit in patients with advanced solid tumors
Source: Cell Res. 2024 Jun 24;34(9):661–4. doi: 10.1038/s41422-024-00990-9 (PMC11369195; doi:10.1038/s41422-024-00990-9)
Supplement: Supplementary file 2 — Supplementary Figure 2 [file 41422_2024_990_MOESM2_ESM.pdf]

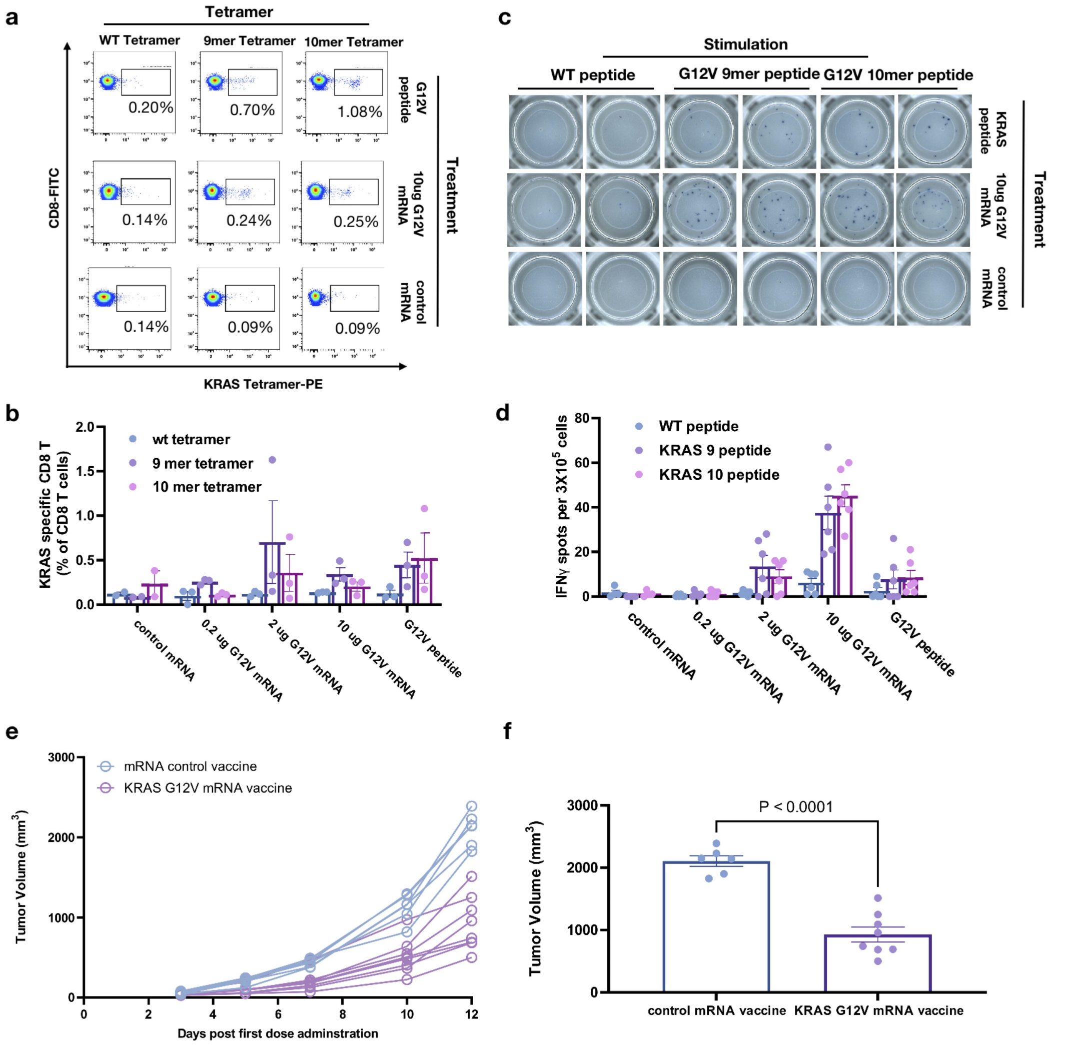

Figure S2. KRAS G12V specific T cell expansion and activation in HLA-A*11:01 mice upon G12V mRNA vaccine treatment. a-b) Tetramer staining of KRAS G12V/HLA-A11:01 specific CD8 T cells in spleen after the treatment of G12V peptide or mRNA vaccine. a) representative flow cytometry plots and b) statistical analysis; c-d) ELISPOT of post-vaccine splenocytes stimulated with KRAS G12V peptides. c) representative ELISPOT picture and d) statistical analysis. e-f) KRAS G12V and HLA-A11:01 overexpressing B16F10 melanoma cells were subcutaneously implanted into HLA-A11:01 mice. After 3 days, 3 doses of KRAS G12V mRNA vaccine or control vaccine (10ug each dose) were given to mice with 5 days of interval between each dose. Tumor growth curve and tumor volume on day 12 after first dose of vaccine are shown in e) and f).
