# Supplementary material for: Combination therapy of KRAS G12V mRNA vaccine and pembrolizumab: clinical benefit in patients with advanced solid tumors
Source: Cell Res. 2024 Jun 24;34(9):661–4. doi: 10.1038/s41422-024-00990-9 (PMC11369195; doi:10.1038/s41422-024-00990-9)
Supplement: Supplementary file 3 — Supplementary Figure 3 [file 41422_2024_990_MOESM3_ESM.pdf]

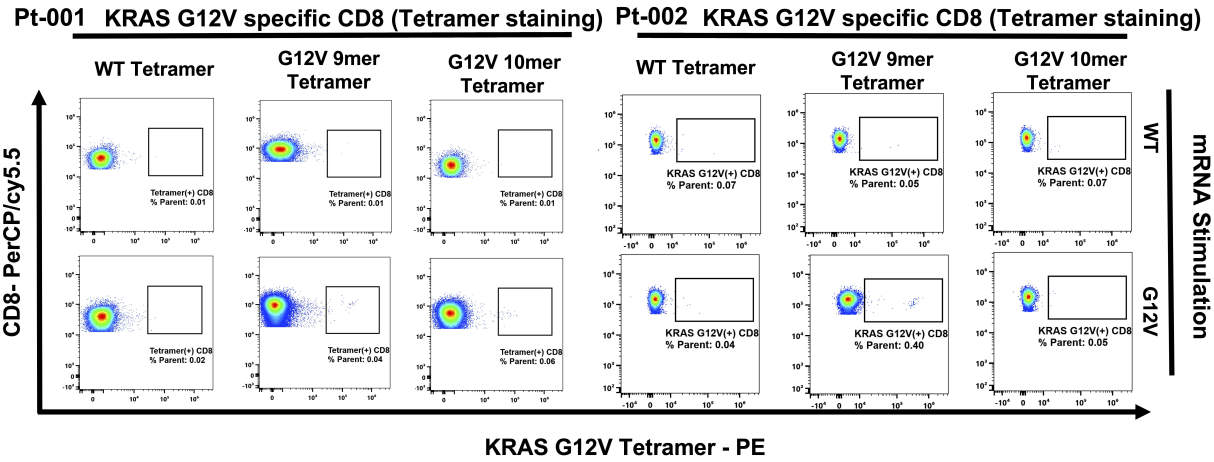

Figure S3. KRAS G12V specific CD8 T cells in post-vaccination PBMCs after being pulsed with KRAS G12V antigen using mRNA (electrophoresis) ex vivo.
