# Supplementary material for: Combination therapy of KRAS G12V mRNA vaccine and pembrolizumab: clinical benefit in patients with advanced solid tumors
Source: Cell Res. 2024 Jun 24;34(9):661–4. doi: 10.1038/s41422-024-00990-9 (PMC11369195; doi:10.1038/s41422-024-00990-9)
Supplement: Supplementary file 4 — Supplementary Figure 4 [file 41422_2024_990_MOESM4_ESM.pdf]

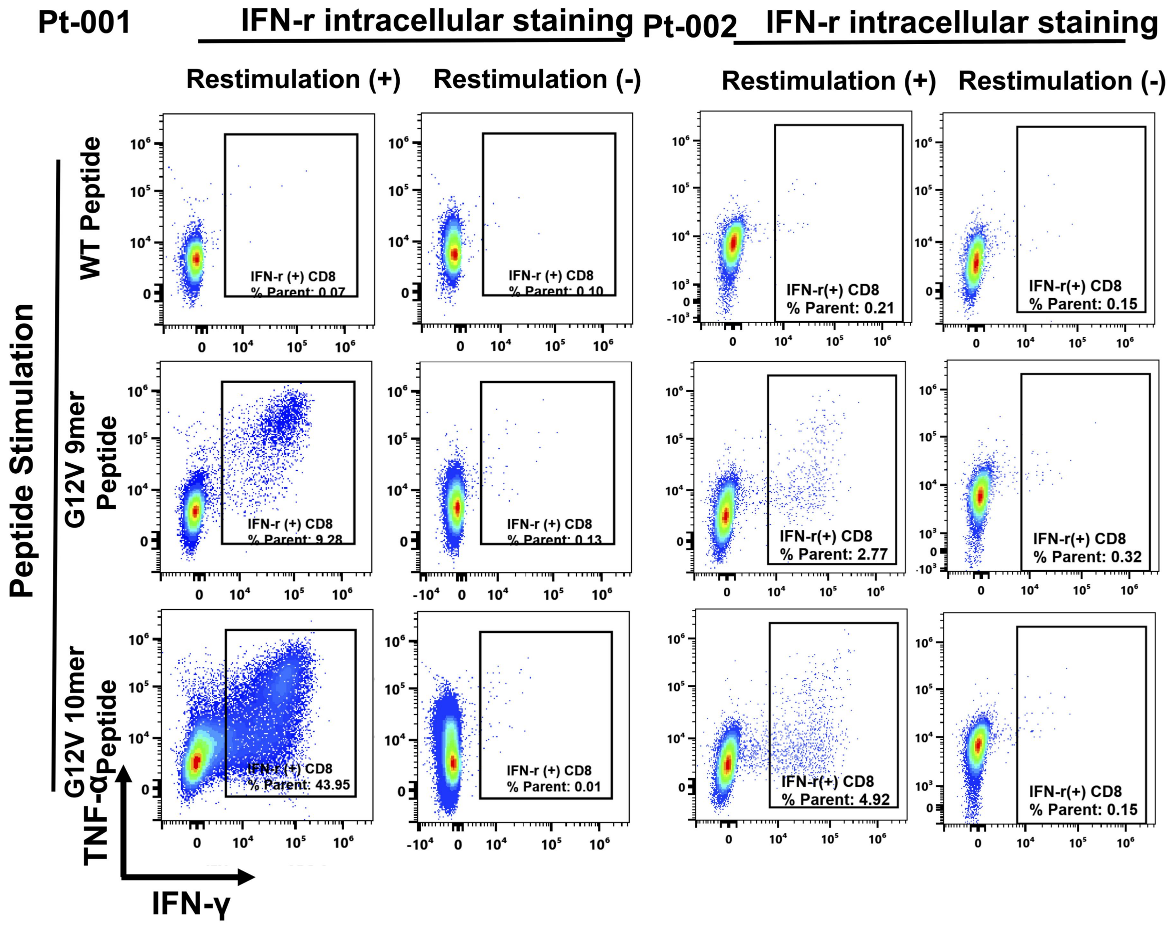

Figure S4: KRAS G12V-specific T cell activation of post-vaccination PBMCs stimulated ex vivo.

CTLs secretion of tumor immunity-related cytokines, including IFN-γ and TNF-α after KRAS peptide ex vivo stimulation.
