# Supplementary material for: Combination therapy of KRAS G12V mRNA vaccine and pembrolizumab: clinical benefit in patients with advanced solid tumors
Source: Cell Res. 2024 Jun 24;34(9):661–4. doi: 10.1038/s41422-024-00990-9 (PMC11369195; doi:10.1038/s41422-024-00990-9)
Supplement: Supplementary file 5 — Supplementary Figure 5 [file 41422_2024_990_MOESM5_ESM.pdf]

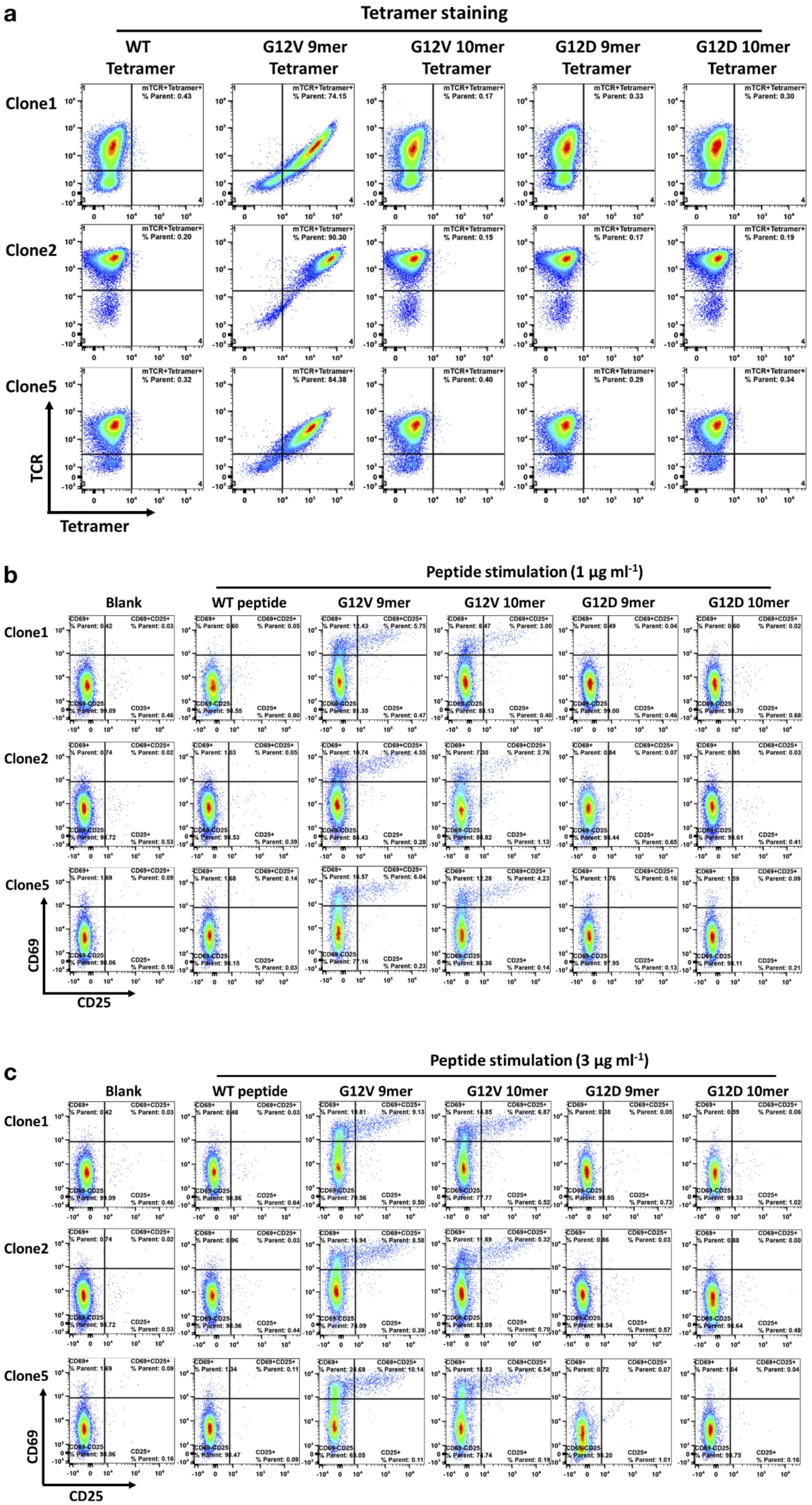
a
Tetramer staining
WT Tetramer
G12V 9mer Tetramer
G12V 10mer Tetramer
G12D 9mer Tetramer
G12D 10mer Tetramer
Clone1
Clone2
Clone5
mTCR+Tetramer+
% Parent: 0.43
% Parent: 74.15
% Parent: 0.17
% Parent: 0.33
% Parent: 0.30
% Parent: 0.20
% Parent: 90.30
% Parent: 0.15
% Parent: 0.17
% Parent: 0.19
% Parent: 0.32
% Parent: 84.38
% Parent: 0.40
% Parent: 0.29
% Parent: 0.34
TCR
Tetramer
b
Peptide stimulation (1 μg ml-1)
Blank
WT peptide
G12V 9mer
G12V 10mer
G12D 9mer
G12D 10mer
CD69+
CD69+CD25+
CD69-CD25-
CD25+
CD69
CD25
c
Peptide stimulation (3 μg ml-1)
Blank
WT peptide
G12V 9mer
G12V 10mer
G12D 9mer
G12D 10mer
CD69
CD25

Figure S5: KRAS G12V-specific TCRs from patient-002 do not react to KRAS G12D mutation. a) the three TCRs were stained with either KRAS G12V or G12D Tetramer staining. b-c) TCR clones co-cultured with PANC-01 pulsed with either KRAS G12V or G12D peptides, and T cell activation markers CD69 and CD25 were measured. WT peptide served as a control.
