# Supplementary material for: Combination therapy of KRAS G12V mRNA vaccine and pembrolizumab: clinical benefit in patients with advanced solid tumors
Source: Cell Res. 2024 Jun 24;34(9):661–4. doi: 10.1038/s41422-024-00990-9 (PMC11369195; doi:10.1038/s41422-024-00990-9)
Supplement: Supplementary file 6 — Supplementary Data S1 [file 41422_2024_990_MOESM6_ESM.doc]

**Table of Contents**

**SUPPLEMENTARY MATERIALS AND METHODS**...................................................... 2

Identification of Neoepitope .................................................................................................... 2

Generation and characterization of mRNA vaccine ................................................................ 2

Ex vivo PBMC stimulation and KRAS G12V-specific T cells detection in humans ...............3

Flow cytometry of immune profile and intracellular staining (ICS) of cytokines of human peripheral blood.........................................................................................................................4

TCR repertoire diversity by TCR sequencing...........................................................................4

COS-7 cell transduction for target mass spectrometry..............................................................4

Targeted mass spectrometry......................................................................................................5

Isolation of HLA peptides.........................................................................................................5

LC-MS/MS................................................................................................................................5

Database search.........................................................................................................................6

Target peptide quantification analysis......................................................................................6

Enzyme-linked immunospot (ELISPOT) assay of mouse splenocytes....................................6

Transduction of Jurkat cells......................................................................................................7

Peptides and peptide pulse and co-culture assay......................................................................7

Inclusion criteria.......................................................................................................................8

**REFERENCES FOR SUPPLEMENTARY APPENDIX** ................................................. 9

**SUPPLEMENTARY MATERIALS AND METHODS**

**Identification of Neoepitope**

Tumor biopsy specimens, serum samples, and hair follicles were collected for next-generation sequencing (NGS) analysis. In brief, DNA and RNA were extracted from the diagnostic sample (QIAGEN) and prepared for sequencing using the KAPA Hyper Prep kit (Illumina). Libraries were sequenced on a HiSeq6000 with PE-150 reads (Illumina), and SNV INDEL variants were called using the variant callers, mutect1(v1.1.7), Mutect2(GATK 4.9.1.0), VarDict (v1.8.2) and scalpel (v0.5.4), only those mutations that were called and marked as PASS by any two softwares were retained and manually inspected in IGV (v2.5.2). Germline and Somatic CNV was analyzed by CNVkit and DECoN. RNA-seq library quality was assessed using FastQC v0.10.0 (http://www.bioinformatics.babraham.ac.uk/projects/fastqc/). Transcript TPM table files were generated by the Hisat2 StringTie and Ballgown pipeline (Pertea, et al. 2016). To predict MHC Type, OptiType and HL*LA were employed to identify the patients’ 4-digit HLA class I and II (HLA-A, HLA-B, HLA-C, HLA-DQA1, HLA-DQB1, HLA-DRB1) type.

NetMHCpan 4.1 tools were applied to predict MHC class I binding of 8 to 11mer mutant peptides to the patients’ HLA-A, HLA-B, and HLA-C alleles for each nonsynonymous and frameshift mutation identified by NGS (Table S4).

**Generation and characterization of mRNA vaccine**

A plasmid encoding the RNA polymerase promoter followed by hAg 5’UTR, with optimized Kozak sequence, and ORF, hAg 3’UTR, and 120nt poly(A) tail were synthesized. The ORF coding for KRAS G12V neo-antigen (amino acid sequence: MTEYKLVVVGAVGVGKSALTIQLIQ) was connected by non-immunogenic glycine/serine linkers (start linker for GGSGGGGSGG, end linker GGSLGGGGSG), containing the signal peptide (amino acid sequence: MRVTAPRTLILLLSGALALTETWAGS) and MHC I trafficking domain (amino acid sequence: IVGIVAGLAVLAVVVIGAVVATVMCRRKSSGGKGGSYSQAASSDSAQGSDVSLTA). The plasmid was overexpressed in E.coli and linearized by BspQI and subjected to in vitro transcription with T7 RNA polymerase in the presence of 8mM ATP, CTP, GTP, UTP, and 8mM Cleancap AG. RNA was purified using Thermo Dynabeads™ MyOne™ Streptavidin C1, and integrity was assessed by Agilent fragment analyzer.

The mRNA was then encapsulated into lipid nanoparticles (LNPs) for in vivo delivery. LNP formulations were prepared using a modified procedure, a method described for siRNA^1^. Briefly, lipids were dissolved in ethanol at molar ratios of 50:10:38.5:1.5 (ionizable lipid: DSPC: cholesterol: PEG-lipid). The lipid mixture was combined with a 50 mm citrate buffer (pH 4.0) containing mRNA at a ratio of 3:1 (aqueous:ethanol) using a microfluidic mixer (Precision Nanosystems, Vancouver, BC). Formulations were filtered against PBS (pH 7.4) by using Amicon Ultra Centrifugal Filters (EMD Millipore, Billerica, MA), passed through a 0.22-mm filter and stored at 4℃ until use. All formulations were tested for particle size, RNA encapsulation, and endotoxin were found to be between 60 to 100 nm in size, with greater than 90% encapsulation and < 5 EU/ml of endotoxin.

**Ex vivo PBMC stimulation and KRAS G12V-specific T cells detection in human**

Peripheral blood mononuclear cells (PBMCs) were isolated using Ficoll-Paque™ (Cytiva) and cultured in RPMI 1640 + 5% human serum (HS) + 1% penicillin/streptomycin at 37℃ and 5% CO_2_ at 10^6^/mL concentration. GM-CSF and IL-4 (both 100ng/ml) were used to differentiate CD14^+^ monocytes into DCs. After 24 hours, additional DC maturation cytokines, including IL-6, IL-1β, TNF-α, LPS, and PGE2. At the same time, KRAS G12V 9 aa or 10 aa peptide (VVGAVGVGK or VVVGAVGVGK) or KRAS WT peptide were added to the culture at a concentration of 1μM. After 24 hours, PBMCs were fed with lymphocyte proliferation cytokines, including IL-2, Il-7, and Il-15 (Peprotech) at 2ng/mL. The culture medium was half-changed every 2-3 days with a total of 3 feedings. On day 10, cells were collected and stained with PE-HLA-A*11:01 tetramer (customized by MBL, Catalog # TS3035, TS3036 and TS3037, MBL) conjugated with KRAS G12V 9mer, 10mer, or WT to detect KRAS G12V-specific T cells using flow cytometry (Cytek Aurora). CD3, CD4, CD8, and 7-AAD live/dead dye were also used in this flow cytometry panel.

**Flow cytometry of immune profile and intracellular staining (ICS) of cytokines of human peripheral blood**

Patients’ whole blood lysed with RBC lysis buffer was incubated with an antibody cocktail to perform the immune profiling. In panel 1, CD3, CD4, CD8, CD19. CD16, CD56, TCRγδ, CD2, CD5, CD7, CD45RO, CD45RA, CD45, TRBC-1 antibodies were used. In panel 2, Cytek® 25-Color Immunoprofiling Assay was used (Cytek, SKU R7-40002). For ICS staining, cells were stimulated with mutant or WT peptide overnight, and Brefeldin A (BFA) was added to the culture system 4 hours before harvesting the cells. ICS was performed using BD Cytofix/Cytoperm™ Fixation/Permeabilization Kit (554714). Antibody cocktails being used include CD3, CD4, CD8, IFN-γ, TNF-α, IL-2, Granzyme B, and LIVE/DEAD™ Fixable Aqua Dead Cell dye. All experiments were using Full Spectrum cytometer from Cytek (3 laser Nothenlight or 5 laser Aurora)

**TCR repertoire diversity by TCR sequencing**

PBMCs from patients were isolated by Ficoll-Paque. RNA was extracted from PBMCs using the RNAeasy Plus Minikit(Qiagen). The TCR library containing TCRα/β sequences were generated using the lab self-developed kit and sequenced on Illumina Novaseq with PE-150 reads. FASTQ files were then processed for TRB and TRA sequence annotation using MiXCR software (v4.3.1).

**COS-7 cell transduction for target mass spectrometry**

COS-7 cells were transduced with the plasmid containing human HLA-A*11:01 followed by GFP Tag. The HLA-I transduction efficiency was validated by measuring GFP-positive cell percentage. GFP-positive cells were then sorted to enrich the HLA-A*11:01 overexpressing cells by using Cytek Aurora CS Sorter (Cytek). After two rounds of sorting, GFP positive percentage cells were enriched to >95% (Data not shown). 1×10^6^ cells were transfected with KRAS G12V or KRAS WT mRNA (40μg/sample) for 24 hours and then harvested for target mass spectrometry.

**Targeted mass spectrometry**

Targeted mass spectrometry analysis was sourced out and performed by Baizhen Biotechnologies, Wuhan, China. Brief steps include isolation of HLA peptide, LC-MS/MS, database search and target peptide quantification analysis. Detailed protocol is described as follows.

**Isolation of HLA peptides**

HLA-peptide complexes were enriched by a commercial sample preparation kit (Baizhen Biotechnologies, # BZNEO-H-1-10) according to the manufacturer manual. In brief, the cell pellet was gently lysed in pre-cold lysis buffer with pipetting, incubated on ice for 60 minutes, and then sonicated for 5 minutes. Cell lysates were collected after centrifugation. A BCA assay was used to measure the protein concentration and clarify the quality of protein extraction. Supernatants were transferred to a new tube for HLA-peptides complexes enrichment and incubated end-over-end at 4°C overnight. HLA-bound peptides were separated and cleaned by a C18 spin column and dried out by a speed vacuum.

**LC-MS/MS**

Peptides were reconstituted by 0.1% Formic Acid in water and separated on a homemade C18 column (150 mm x 75 μm i.d., 1.9 Å particle size; Dr. Maisch GmbH) by an Easy-nLC 1200 HPLC at 300nL/min with a 30 min gradient of 2-100% Acetonitrile (with 0.1% formic acid). MS data were acquired by an Orbitrap Fusion Tribrid Mass Spectrometer (Thermo Scientific) in a target PRM (parallel reaction monitor) acquisition mode with scan range of 300–800 m/z. MS/MS was performed via higher energy collisional dissociation fragmentation. Full MS and MS/MS scans were acquired at a resolution of 120,000 and 30,000, respectively.

**Database search**

The acquired MS/MS data were analyzed against a Uniprot *Cercopithecus aethiops* database (18422 Entries) with two KRAS sequences (WT: MTEYKLVVVGAGGVGKSALTIQLIQ, MT: MTEYKLVVVGAVGVGKSALTIQLIQ) by PEAKS online v1.8 peptidome workflow. Tolerance of precursor mass and fragment mass were set to 10 p.p.m. and 0.02 Da. Oxidation of methionine (+15.9949 Da) was set as variable modifications. The Enzyme was set as none. A contaminant database containing 204 proteins was added to exclude the contaminant peptides hint. FDR of the identified peptides was set at 1%.

**Target peptide quantification analysis**

By database search, the targeted KRAS peptide identification information, including retention time, was obtained. The chromatography peak of the identified peptide form in each sample was extracted and integrated by Xcalibur (v4.5, Thermo Scientific). The MS1 peak area was recorded as quantification information.

The PRM setting of target peptide sequences is listed below.

| peptide | m/z | charge |
| --- | --- | --- |
| VVVGAVGVGK | 442.7818 | 2+ |
| VVGAVGVGK | 393.2476 | 2+ |
| VVVGAGGVGK | 421.7584 | 2+ |
| VVGAGGVGK | 372.2241 | 2+ |

**Enzyme-linked immunospot (ELISPOT) assay of mouse splenocytes**

Mouse splenocytes were isolated and were then transferred to ELISpot Plus: Mouse IFN-γ (HRP) plates pre-coated with IFN-γ antibodies (MebTech, 3321-4HPW-2) at the concentration of 0.3×10^6^ /well. At the same time, cells were stimulated with KRAS G12V 9mer, 10mer, or WT peptides in 1640 medium with 10% FBS for 18-24 hours. After incubation, the plate used for IFN-γ detection based on the manufacture protocol.

**Transduction of Jurkat cells**

Jurkat cells equipped with constitutive human CD8 expression by CRISPR-Cas9 HDR targeting the CCR5 safe harbor locus was described previously^2^. For transduction of Jurkat cells, a total of 0.5x10^6^ Jurkat cells were plated per well in 24-well plate, equal amount of lentiviral supernatant was applied to the cells in the presence of 8 μg /ml polybrene. Cells were then centrifuged for 90 minutes at 1000g and incubated at 37°C for 2 hours. Transduction efficiency was determined by expression of eGFP at 3 days for Jurkat cells.

**Peptides and peptide pulse and co-culture assay**

Peptides were generated by custom peptide synthesis (GL Biochem, Shanghai), resuspended at 10 mg/ml in DMSO and placed at -80℃ for prolonged storage. For peptide pulsing, PANC-01 cells were harvested and washed twice in serum-free RPMI 1640 (SF-RPMI). Peptides were diluted to 10 mg/ml in SF-RPMI (or to concentrations indicated in figure legends) and the solution was used to re-suspend cells at 1x10^6^ cells /ml. Cells were incubated for 90 min at 37℃, 5% CO2, washed once with SF-RPMI, re-suspended in complete media and added to co-culture wells. Jurkat cells with constitutive TCR expression (TCR-Jurkat) were harvested, pelleted by centrifugation, and re-suspended in fresh complete media at 1x10^6^ cells/ml. 1x10^5^ TCR-Jurkat cells (100 μL) were seeded in wells of a U-bottom 96-well plate. PANC-01 cells pulsed by different peptides were adjusted to 1x10^6^ cells/ml in complete media and 5x10^4^ cells (50 μL) were added to each well. Anti-human CD28 antibody (clone CD28.2, BD bioscience, #556620) was added at a final concentration of 1 mg/ml for co-stimulation of all samples (including negative controls) and plates were incubated overnight at 37℃, 5% CO2. The next day, expression of CD69 and CD25 in TCR-Jurkat cells was assessed by flow cytometry.

**Inclusion criteria**

Patients who have advanced or metastatic diseases and fail or cannot handle first-line treatment can potentially become candidates for this study. In more detail, we evaluate patients based on the following criteria.

1. Subjects must willingly provide written informed consent, demonstrating their ability to adhere to the study protocol as determined by the investigator.
2. Subjects must be ≥ 18 years of age at the time of informed consent, irrespective of gender.
3. Subjects must have histologically or cytologically confirmed locally advanced, recurrent, or metastatic solid tumors within the past 6 months. They should have either failed standard treatment or been deemed unsuitable.
4. Gene sequencing should reveal the absence of copy number variations or loss of heterozygosity in HLA-related genes and chromosomal regions.
5. Advanced or metastatic lesions, confirmed by immunohistochemistry, along with cryopreserved tissues/cells sufficient for WES and RNA sequencing, must be available. Bioinformatics analysis should predict the presence of at least one antigen effectively presented by self-HLA.
6. Subjects should have a life expectancy of ≥ 4 months.
7. Measurable disease per RECIST 1.1, as assessed by the local site investigator/radiologist, is required. Lesions previously irradiated are considered measurable if progression is evident.

**REFERENCES FOR SUPPLEMENTARY APPENDIX**

1 Chen S, Tam YYC, Lin PJC, Sung MMH, Tam YK, Cullis PR. Influence of particle size on the in vivo potency of lipid nanoparticle formulations of siRNA. *J Control Release* 2016; **235**:236-244.

2 Sather BD, Romano Ibarra GS, Sommer K *et al.* Efficient modification of CCR5 in primary human hematopoietic cells using a megaTAL nuclease and AAV donor template. *Sci Transl Med* 2015; **7**:307ra156.
