# Supplementary material for: Combination therapy of KRAS G12V mRNA vaccine and pembrolizumab: clinical benefit in patients with advanced solid tumors
Source: Cell Res. 2024 Jun 24;34(9):661–4. doi: 10.1038/s41422-024-00990-9 (PMC11369195; doi:10.1038/s41422-024-00990-9)
Supplement: Supplementary file 7 — Supplementary Table 1 [file 41422_2024_990_MOESM7_ESM.pdf]

Table S1. Somatic Mutations Identified by Whole-Exome and Transcriptome Sequencing of Patient-001.

Gene Symbol	Transcript ID	cDNA Change	Cov AD	AF DNA(%)	AF RNA(%)	TPM
LMO4	NM_001369491.1	c.169C>G	36,7	16.28	46%	31.440
FMNL2	NM_052905.4	c.2606A>G	67,9	11.84	0%	7.582
TRAK1	NM_001042646.3	c.1654C>T	42,11	20.75	50%	158.199
HGFAC	NM_001297439.2	c.1918C>T	212,40	15.87	0%	0
PIK3R1	NM_181523.3	c.202G>C	90,21	18.92	20%	5.581
ARHGEF28	NM_001080479.2	c.4976C>T	57,15	20.83	0%	7.653
NKAPL	NM_001007531.3	c.1116_1117delTA	43,8	15.69	0%	0
FAM184A	NM_024581.6	c.2292G>T	114,11	8.8	0%	0.377
HIBADH	NM_152740.4	c.512T>A	107,18	14.4	23%	12.609
KCNU1	NM_001031836.3	c.3004C>T	114,15	11.63	0%	0
SMC2	NM_001042550.2	c.3394C>T	89,13	12.75	100%	1.006
COL17A1	NM_000494.4	c.4357G>A	53,9	14.52	0%	17.739
INPP5F	NM_014937.4	c.631delT	59,7	10.61	0%	4.117
ATM	NM_000051.3	c.2965delA	102,17	14.29	0%	2.074
ATM	NM_000051.3	c.6908dupA	56,12	17.65	0%	2.074
KRAS	NM_001369786.1	c.35G>T	74,10	11.9	77%	7.853
ERBB3	NM_001982.3	c.889G>T	39,16	29.09	100%	9.053
ATXN2	NM_001372574.1	c.1112A>T	66,13	16.25	0%	9.273
NBEA	NM_015678.4	c.8485A>T	55,24	30.38	0%	0.401
SLITRK1	NM_001281503.2	c.1572A>G	41,7	14.58	0%	0
MYO16	NM_001198950.3	c.5230A>T	216,29	11.84	0%	0.348
CHD8	NM_001170629.2	c.3368_3369delTT	33,9	21.43	14%	9.113
RPUSD2	NM_152260.3	c.725C>G	59,19	24.36	0%	2.025
UNC13C	NM_001080534.2	c.2927C>T	58,13	18.31	0%	0.108
CLCN7	NM_001287.6	c.1081G>A	51,13	20.31	20%	10.548
SPATA22	NM_001170695.1	c.8G>A	33,6	15.38	0%	0
RAI1	NM_030665.4	c.1828G>A	42,9	17.65	24%	12.232
P3H4	NM_006455.3	c.1217A>G	63,6	8.7	0%	2.491
ZNF490	NM_020714.3	c.1255G>A	70,16	18.6	0%	2.487
ZNF284	NM_001037813.4	c.615T>G	56,10	15.15	0%	1.177
IL4I1	NM_001258017.2	c.578G>A	48,8	14.29	0%	8.507
SYCP2	NM_014258.4	c.3948dupA	83,6	6.59	0%	0
MIR7-3HG	NR_027148.1	n.206C>T	47,7	12.96	0%	0
CYP4F24P	NR_033864.1	n.994G>A	78,18	18.75	0%	0
XIST	NR_001564.2	n.3187G>A	63,10	13.7	0%	6.963
