# Supplementary material for: Combination therapy of KRAS G12V mRNA vaccine and pembrolizumab: clinical benefit in patients with advanced solid tumors
Source: Cell Res. 2024 Jun 24;34(9):661–4. doi: 10.1038/s41422-024-00990-9 (PMC11369195; doi:10.1038/s41422-024-00990-9)
Supplement: Supplementary file 8 — Supplementary Table 2 [file 41422_2024_990_MOESM8_ESM.pdf]

Table S2. Somatic Mutations Identified by Whole-Exome and Transcriptome Sequencing of Patient-002.

Gene Symbol	Transcript ID	cDNA Change	Cov AD	AF DNA(%)	AF RNA(%)	TPM
AGTRAP	NM_020350.5	c.382C>T	102,7	0.0642	0.1298	14.685493
DDX20	NM_007204.5	c.158C>T	69,6	0.08	0.0000	8.702319
PKLR	NM_000298.6	c.378C>A	131,37	0.2202	0.0000	0
FCRL3	NM_001320333.2	c.827C>A	111,8	0.0672	0.0000	3.375108
DUSP27	NM_001080426.3	c.2833G>T	88,12	0.12	0.0000	0.101741
ADCY10	NM_018417.6	c.3928G>A	153,7	0.0437	0.0000	0.208169
TNR	NM_003285.3	c.1121C>A	100,16	0.1379	0.0000	0.014855
NFASC	NM_001005388.2	c.2724dupT	108,14	0.1148	0.0000	6.226189
SLC30A1	NM_021194.3	c.191C>T	164,9	0.052	0.1441	4.017916
DNAH14	NM_001367479.1	c.12659T>G	37,8	0.1778	0.0000	4.936102
MTR	NM_000254.2	c.1438G>A	66,8	0.1081	0.0687	36.874161
OR2G2	NM_001001915.1	c.4G>T	61,6	0.0896	0.0000	0.10915
OR1C1	NM_012353.2	c.884G>A	69,6	0.08	0.0000	0
KCNF1	NM_002236.5	c.1382C>A	147,9	0.0577	0.0000	0.09027
IL37	NM_014439.3	c.384A>T	114,8	0.0656	0.0909	6.495797
GOLGA4	NM_001172713.2	c.5995G>A	27,8	0.2162	0.1739	57.216335
DCAF1	NM_001349168.2	c.3771A>T	68,7	0.0933	0.0000	18.335356
CADM2	NM_153184.3	c.385C>A	69,4	0.0548	0.0000	0.037585
CBLB	NM_001321786.1	c.739A>G	76,7	0.0843	0.0554	43.47554
KLHL6	NM_130446.4	c.1808C>A	138,16	0.1039	0.0000	5.497872
HMX1	NM_018942.3	c.805G>T	79,13	0.1413	0.0000	0
INPP4B	NM_001331040.1	c.1593C>A	101,9	0.0818	0.4124	33.786911
EGFLAM	NM_001205301.2	c.184G>T	69,7	0.0921	0.0000	4.825303
ACOT12	NM_130767.3	c.731G>T	79,12	0.1319	0.0000	0.033409
FBN2	NM_001999.4	c.109C>A	124,7	0.0534	0.0000	4.007003
CHSY3	NM_175856.5	c.677T>C	159,12	0.0694	0.0667	4.168712
REEP2	NM_001271803.2	c.671C>T	146,18	0.1098	0.1875	1.167137
H3C6	NM_003532.2	c.7C>A	148,8	0.0513	0.0000	0
EYS	NM_001292009.1	c.2092G>A	71,6	0.0779	0.0000	0.638845
NT5DC1	NM_152729.3	c.452A>G	19,6	0.24	0.1863	10.456548
PRKAR1B	NM_001164758.1	c.825G>T	133,14	0.0952	0.1692	8.84372
INTS1	NM_001080453.3	c.2528G>T	72,6	0.0769	0.1166	20.693308
ZNF316	NM_001278559.2	c.2216G>T	145,17	0.1049	0.0000	0.046484
NUP42	NM_001370443.1	c.284_294del	78,4	0.0488	0.0000	19.295292
WIPF3	NM_001080529.2	c.1199C>A	102,7	0.0642	0.0000	0.070046
ABCA13	NM_152701.5	c.3341C>A	66,6	0.0833	0.1667	0.767964
ZNF679	NM_153363.3	c.17G>A	101,6	0.0561	0.0000	0
FZD9	NM_003508.3	c.1672C>A	191,17	0.0817	0.0000	0
PCLO	NM_033026.6	c.11291A>G	99,15	0.1316	0.3000	4.114597

ABCB1	NM_001348945.1	c.2494delC	43,7	0.14	0.0000	6.711885
TRRAP	NM_001244580.1	c.7414G>A	134,13	0.0884	0.0964	36.848129
OR6V1	NM_001001667.1	c.217A>G	112,18	0.1385	0.0000	0
TMEM176B	NM_001362691.1	c.538T>C	106,14	0.1167	0.0011	230.016739
NEIL2	NM_001135746.3	c.269G>T	116,10	0.0794	0.0870	6.3938
ANK1	NM_001142446.2	c.440C>G	76,8	0.0941	0.0000	1.002439
RECQL4	NM_004260.3	c.2702A>C	152,19	0.1111	0.1250	3.46006
TAF1L	NM_153809.2	c.3088G>T	143,21	0.128	0.0000	0.173484
NPR2	NM_003995.3	c.2372+1G>C	115,7	0.0574	0.0000	32.702236
CIZ1	NM_001257975.1	c.1626A>T	168,15	0.082	0.0377	42.535984
SARDH	NM_001134707.2	c.2431G>T	156,12	0.0714	0.0000	2.328412
SLC25A28	NM_031212.4	c.959G>T	110,8	0.0678	0.1437	24.967113
TLX1	NM_005521.4	c.858C>A	142,11	0.0719	0.0000	0
AS3MT	NM_020682.4	c.686G>A	95,7	0.068	0.0154	3.838742
IRF7	NM_004031.4	c.1534C>A	115,10	0.08	0.1494	9.078856
TRPM5	NM_014555.3	c.2167C>T	107,10	0.0855	0.0000	0
GLYATL1	NM_080661.4	c.618T>A	31,6	0.1622	0.0000	0
NUMA1	NM_006185.3	c.1346A>C	116,13	0.1008	0.0580	73.678795
USP35	NM_020798.4	c.2899A>G	123,11	0.0821	0.0000	3.282557
KRAS	NM_001369786.1	c.35G>T	46,5	0.098	0.4713	15.035834
LRRK2	NM_198578.4	c.7355delG	59,9	0.1324	0.0099	333.703888
COL2A1	NM_001844.5	c.2911C>T	160,16	0.0909	0.0000	0.035041
H1-7	NM_181788.1	c.243G>T	145,22	0.1317	0.0000	0.550036
XPOT	NM_007235.6	c.2341G>A	57,6	0.0952	0.2381	44.777714
RIMBP2	NM_001351226.2	c.2002T>G	64,7	0.0986	0.0000	0
VWA8	NM_015058.2	c.1273T>G	82,13	0.1368	0.0068	22.68552
MLNR	NM_001507.1	c.463G>T	106,10	0.0862	0.0000	0
ATP7B	NM_000053.4	c.4246G>T	142,10	0.0658	0.0800	8.5521
UTP14C	NM_021645.6	c.1030G>C	113,10	0.0813	0.1372	26.140955
CHD8	NM_001170629.2	c.5943dupT	124,9	0.0677	0.0400	49.525265
LTB4R	NM_001143919.3	c.857C>T	108,8	0.069	0.2000	10.441017
RTN1	NM_021136.3	c.1922A>G	86,9	0.0947	0.0000	4.474306
LRRC9	NM_001355272.2	c.2555C>T	80,9	0.1011	0.0000	0
NPAP1	NM_018958.3	c.585G>T	112,16	0.125	0.0000	0.026101
TJP1	NM_001301025.3	c.3413A>T	81,6	0.069	0.1497	82.499557
UACA	NM_018003.4	c.3138T>A	70,6	0.0789	0.0000	42.285172
PDIA2	NM_006849.4	c.711C>A	109,13	0.1066	0.0000	0.446837
CLCN7	NM_001287.6	c.2340delG	143,11	0.0714	0.0706	89.666779
ZNF263	NM_005741.5	c.880G>T	38,6	0.1364	0.3333	6.194598
ADCY9	NM_001116.4	c.2732A>T	120,8	0.0625	0.0918	8.975513
TVP23A	NM_001079512.4	c.394_395insA	88,8	0.0833	0.0000	0.41516
C16orf54	NM_175900.4	c.343G>T	74,10	0.119	0.0000	1.232111
SRCAP	NM_006662.3	c.5891A>G	151,15	0.0904	0.0886	45.012852
FOXF1	NM_001451.3	c.844G>A	125,11	0.0803	0.0000	3.808731

ZNF18	NM_001303281.2	c.1385A>T	48,7	0.1273	0.0909	3.78036
KRT20	NM_019010.3	c.371T>C	67,8	0.1067	0.0000	0
KRTAP9-1	NM_001190460.1	c.425C>A	172,17	0.0899	0.0000	0
STAT5B	NM_012448.4	c.1442C>T	104,6	0.0545	0.0637	34.251652
NARF	NM_031968.2	c.115G>C	104,10	0.0877	0.1500	41.205009
CDH7	NM_001362438.2	c.1365G>A	89,8	0.0825	0.0000	0
TPGS1	NM_033513.3	c.790C>T	72,10	0.122	0.0000	0.538985
CACNA1A	NM_023035.3	c.6104C>A	105,6	0.0541	0.0000	0.087568
ASF1B	NM_018154.3	c.266_307del	156,5	0.0311	0.0000	1.65109
UBA2	NM_005499.3	c.22C>T	77,7	0.0833	0.0000	44.563416
CPT1C	NM_001199752.2	c.1915G>A	147,11	0.0696	0.0000	4.175367
SIGLEC7	NM_014385.3	c.641G>T	81,15	0.1562	0.0000	13.083031
NLRP12	NM_001277126.1	c.353T>C	102,12	0.1053	0.0000	2.410324
PEG3	NM_001369717.1	c.3823C>T	99,9	0.0833	0.0000	2.30054
DOK5	NM_018431.5	c.605G>A	53,7	0.1167	0.0000	1.612555
TMPRSS15	NM_002772.3	c.2152C>A	91,8	0.0808	0.0000	0.097835
DEPDC5	NM_001242896.3	c.679G>A	105,5	0.0455	0.0000	23.703848
RTCB	NM_014306.5	c.740delG	115,8	0.065	0.0029	33.175888
DNAJB7	NM_145174.2	c.232A>C	82,10	0.1087	0.0714	0
TCF20	NM_005650.3	c.646A>C	144,7	0.0464	0.0446	31.699396
NLGN4X	NM_001282145.2	c.755G>T	56,9	0.1385	0.0000	3.01662
MAGEC2	NM_016249.4	c.200A>G	45,17	0.2742	0.0000	0
F8	NM_000132.3	c.3500A>G	30,8	0.2105	0.0000	8.731336
