# Supplementary material for: Combination therapy of KRAS G12V mRNA vaccine and pembrolizumab: clinical benefit in patients with advanced solid tumors
Source: Cell Res. 2024 Jun 24;34(9):661–4. doi: 10.1038/s41422-024-00990-9 (PMC11369195; doi:10.1038/s41422-024-00990-9)
Supplement: Supplementary file 9 — Supplementary Table 3 [file 41422_2024_990_MOESM9_ESM.pdf]

Table S3. PD-1/PD-L1/PD-L2 expression by whole exon sequencing and transcriptome sequencing.

Ensembl ID	Gene Symbol	Patient-001	Patient-002
ENSG00000188389	PDCD1(PD-1)	0	0.997959
ENSG00000120217	CD274(PD-L1)	0.410037	14.18261
ENSG00000197646	PDCD1LG2(PD-L2)	0.606788	6.31238
ENSG00000111640	GAPDH	3372.454	1084.546
