# Supplementary material for: Combination therapy of KRAS G12V mRNA vaccine and pembrolizumab: clinical benefit in patients with advanced solid tumors
Source: Cell Res. 2024 Jun 24;34(9):661–4. doi: 10.1038/s41422-024-00990-9 (PMC11369195; doi:10.1038/s41422-024-00990-9)
Supplement: Supplementary file 10 — Supplementary Table 4 [file 41422_2024_990_MOESM10_ESM.pdf]

Table S4. HLA-I and HLA-II alleles are determined by next-generation sequencing using the algorithm as described[1].

Patient ID	HLA-I					
	A1	A2	B1	B2	C1	C2
Patient -001	A*11:01	A*33:03	B*13:01	B*44:03	C*03:04	C*14:03
Patient -002	A*11:01	A*11:01	B*13:01	B*38:02	C*03:04	C*07:02
	HLA-II					
	DRB1	DRB1	DQA1	DQA1	DQB1	DQB1
Patient -001	DRB1*12:02	DRB1*13:02	DQA1*01:02	DQA1*06:01	DQB1*03:01	DQB1*06:04
Patient -002	DRB1*12:02	DRB1*15:01	DQA1*06:01	DQA1*01:02	DQB1*06:01	DQB1*12:02

1. Dilthey AT, Mentzer AJ, Carapito R et al. HLA*LA-HLA typing from linearly projected graph alignments. *Bioinformatics (Oxford, England)* 2019; 35:4394-4396.
