# Supplementary material for: Combination therapy of KRAS G12V mRNA vaccine and pembrolizumab: clinical benefit in patients with advanced solid tumors
Source: Cell Res. 2024 Jun 24;34(9):661–4. doi: 10.1038/s41422-024-00990-9 (PMC11369195; doi:10.1038/s41422-024-00990-9)
Supplement: Supplementary file 11 — Supplementary Table 5 [file 41422_2024_990_MOESM11_ESM.pdf]

Table S5. Predicted Neoantigens for Patient 001 by NetMHCPan 4.1 tools

Chr	Allelepos	Ref	Alt	GeneName:RefSeqID	HLA	Peptide	%Rank_EL	Aff(nM)	BindLevel
chr11	1.08E+08	-	A	ATM:NM_000051	HLA-B*44:03	CSEQSQPKTY	0.218	586.26	SB
chr11	1.08E+08	-	A	ATM:NM_000051	HLA-B*44:03	LCSEQSQPKTY	0.268	4249.91	SB
chr11	1.08E+08	-	A	ATM:NM_000051	HLA-B*44:03	QLCSEQSQPKTY	0.123	1753.86	SB
chr11	1.08E+08	-	A	ATM:NM_000051	HLA-B*44:03	SEQSQPKTY	0.002	66.22	SB
chr11	1.08E+08	-	A	ATM:NM_000051	HLA-B*13:01	EQSQPKTYI	0.296	3611.36	SB
chr11	1.08E+08	-	A	ATM:NM_000051	HLA-B*44:03	SEQSQPKTYI	0.257	754.7	SB
chr11	1.08E+08	-	A	ATM:NM_000051	HLA-A*33:03	QSQPKTYIHR	0.352	477.17	SB
chr11	1.08E+08	-	A	ATM:NM_000051	HLA-B*13:01	RMSEGLWQL	0.009	36.78	SB
chr11	1.08E+08	-	A	ATM:NM_000051	HLA-A*33:03	EGLWQLVSR	0.145	71.62	SB
chr11	1.08E+08	-	A	ATM:NM_000051	HLA-A*33:03	QLVSRNVLR	0.111	42.16	SB
chr11	1.08E+08	-	A	ATM:NM_000051	HLA-A*11:01	LVSRNVLRK	0.184	33.36	SB
chr12	25398284	C	A	KRAS:NM_033360	HLA-A*11:01	VVGAVGVGK	0.128	38.76	SB
chr12	25398284	C	A	KRAS:NM_033360	HLA-A*11:01	VVVGAVGVGK	0.447	68.99	SB
chr12	56482341	G	T	ERBB3:NM_001982	HLA-A*33:03	VVYQTSCVR	0.493	176.83	SB
chr12	1.12E+08	T	A	ATXN2:NM_001372574	HLA-C*03:04	SMPSRSTSL	0.395	405.74	SB
chr12	1.12E+08	T	A	ATXN2:NM_001372574	HLA-C*14:03	SMPSRSTSL	0.185	29.08	SB
chr14	21871761	AA	-	CHD8:NM_020920	HLA-C*14:03	SFSRQTGSY	0.209	42.69	SB
chr16	1505152	C	T	CLCN7:NM_001287	HLA-A*33:03	DLSSPGLINFR	0.34	231.96	SB
chr16	1505152	C	T	CLCN7:NM_001287	HLA-A*33:03	SSPGLINFR	0.326	119.38	SB
chr17	17698090	G	A	RAI1:NM_030665	HLA-B*13:01	AQEDLASKI	0.05	585.27	SB
chr17	17698090	G	A	RAI1:NM_030665	HLA-B*13:01	AQEDLASKIL	0.489	2586.94	SB
chr17	17698090	G	A	RAI1:NM_030665	HLA-B*44:03	QEDLASKIL	0.282	1746.95	SB
chr17	39959613	T	C	P3H4:NM_006455	HLA-B*44:03	AEFEGGGDY	0.068	59.55	SB

chr17	39959613	T	C	P3H4:NM_006455	HLA-B*44:03	SDAEFEGGGDY	0.456	986.91	SB
chr2	1.53E+08	A	G	FMNL2:NM_052905	HLA-A*11:01	YISSVVKEK	0.281	137.73	SB
chr5	67522705	G	C	PIK3R1:NM_181523	HLA-B*44:03	GERGHFPGTY	0.359	675.27	SB
chr5	67522705	G	C	PIK3R1:NM_181523	HLA-C*14:03	HFPGTYVEY	0.016	12.17	SB
chr7	27582692	A	T	HIBADH:NM_152740	HLA-C*03:04	AARSGNHTF	0.159	170.78	SB
