# Supplementary material for: Combination therapy of KRAS G12V mRNA vaccine and pembrolizumab: clinical benefit in patients with advanced solid tumors
Source: Cell Res. 2024 Jun 24;34(9):661–4. doi: 10.1038/s41422-024-00990-9 (PMC11369195; doi:10.1038/s41422-024-00990-9)
Supplement: Supplementary file 12 — Supplementary Table 6 [file 41422_2024_990_MOESM12_ESM.pdf]

Table S6. Predicted Neoantigens for Patient Patient002 by NetMHCPan 4.1 tools

Chr	Allelepos	Ref	Alt	GeneName:RefSeqID	HLA	Peptide	%Rank_EL	Aff(nM)	BindLevel
chr12	25398284	C	A	KRAS:NM_033360	HLA-A*11:01	VVGAVGVGK	0.128	38.76	SB
chr12	25398284	C	A	KRAS:NM_033360	HLA-A*11:01	VVVGAVGVGK	0.447	68.99	SB
chr14	21862010	-	A	CHD8:NM_020920	HLA-C*03:04	STAAPAVYL	0.214	75.95	SB
chr14	60074054	T	C	RTN1:NM_021136	HLA-A*11:01	ATISFRICK	0.008	4.13	SB
chr14	60074054	T	C	RTN1:NM_021136	HLA-A*11:01	SATISFRICK	0.257	8.09	SB
chr15	30011212	T	A	TJP1:NM_001355015	HLA-C*03:04	LTYEPQLPF	0.141	16.32	SB
chr16	10867228	-	T	TVP23A:NM_001318873	HLA-B*38:02	CHHDMDCVF	0.303	387.06	SB
chr16	10867228	-	T	TVP23A:NM_001318873	HLA-B*38:02	HHDMDCVFF	0.077	376.7	SB
chr16	86545019	G	A	FOXF1:NM_001451	HLA-A*11:01	ALNSGTSYIK	0.456	64.37	SB
chr17	11881539	T	A	ZNF18:NM_001303281	HLA-A*11:01	RTHTGEMPCK	0.234	37.64	SB
chr17	34163214	G	T	TAF15:NM_139215	HLA-C*03:04	VSTDQVVEF	0.263	386.45	SB
chr17	34163214	G	T	TAF15:NM_139215	HLA-A*11:01	STDQVVEFFK	0.251	10.15	SB
chr17	39041067	A	G	KRT20:NM_019010	HLA-C*07:02	AYYRQTEEL	0.09	208.42	SB
chr17	42940238	T	A	EFTUD2:NM_001258353	HLA-C*03:04	YSSDDGVQF	0.136	59.93	SB
chr19	34919359	C	T	UBA2:NM_005499	HLA-C*03:04	LSRGLSREL	0.401	306.69	SB
chr19	50215114	G	A	CPT1C:NM_152359	HLA-C*03:04	VAVDKHQTL	0.001	4.78	SB
chr19	50215114	G	A	CPT1C:NM_152359	HLA-C*03:04	VAVDKHQTLL	0.19	214.27	SB
chr19	50215114	G	A	CPT1C:NM_152359	HLA-A*11:01	AVDKHQTLLK	0.087	27.73	SB
chr19	54318179	A	G	NLRP12:NM_001277129	HLA-A*11:01	EVSPVTPRK	0.149	155.51	SB
chr2	1.14E+08	A	T	IL37:NM_173203	HLA-A*11:01	HSHPSLQLKK	0.178	113.94	SB
chr2	1.14E+08	A	T	IL37:NM_173203	HLA-A*11:01	GHSHPSLQLK	0.196	115.3	SB
chr2	1.14E+08	A	T	IL37:NM_173203	HLA-A*11:01	HSHPSLQLK	0.051	52.99	SB
chr20	53226932	G	A	DOK5:NM_01843	HLA-B*13:01	RMYETGEGL	0.066	141.8	SB
chr20	53226932	G	A	DOK5:NM_01843	HLA-B*13:01	RMYETGEGLF	0.169	129.9	SB

chr22	32188073	G	A	DEPDC5:NM_001369902	HLA-C*07:02	FYNAKSVDEF	0.428	322.04	SB
chr3	1.05E+08	T	C	CBLB:NM_001321799	HLA-C*03:04	MALKSAIDL	0.24	18.27	SB
chr4	1.43E+08	G	T	INPP4B:NM_001331040	HLA-C*07:02	KRLNCIIAM	0.279	220.77	SB
chr5	78919199	C	A	TENT2:NM_001349549	HLA-C*07:02	RRYSMTPLF	0.054	84.63	SB
chr5	82835089	CC	AA	VCAN:NM_004385	HLA-A*11:01	GTVSTNFPK	0.031	5.24	SB
chr5	82835089	CC	AA	VCAN:NM_004385	HLA-A*11:01	SGTVSTNFPK	0.212	6.65	SB
chr6	43466765	A	C	TJAP1:NM_001350567	HLA-A*11:01	AAPAKTPYRK	0.496	161.38	SB
chr7	618959	C	A	PRKAR1B:NM_001164762	HLA-B*38:02	VHFEDGEKI	0.015	475.01	SB
chr7	81594960	C	T	CACNA2D1:NM_000722	HLA-B*13:01	ILNDGGFLL	0.269	393.66	SB
chr7	87170708	G	-	ABCB1:NM_001348944	HLA-C*03:04	FSFRVSHL	0.266	149.52	SB
chr7	87170708	G	-	ABCB1:NM_001348944	HLA-C*07:02	FRVSHLAKL	0.039	51.66	SB
chr7	87170708	G	-	ABCB1:NM_001348944	HLA-C*03:04	LALELFLLL	0.349	73.44	SB
chr7	98565244	G	A	TRRAP:NM_003496	HLA-A*11:01	NSMKRRVYK	0.294	20.38	SB
chr7	98565244	G	A	TRRAP:NM_003496	HLA-C*07:02	RRVYKRLLY	0.166	236.55	SB
chr8	11637237	G	T	NEIL2:NM_001349441	HLA-A*11:01	KQVVEPSGQK	0.192	152.86	SB
chr8	11637237	G	T	NEIL2:NM_001349441	HLA-A*11:01	QVVEPSGQK	0.116	125.29	SB
chr8	1.46E+08	T	G	RECQL4:NM_004260	HLA-C*03:04	VCMGHARAL	0.357	208.26	SB
chr8	1.46E+08	T	G	RECQL4:NM_004260	HLA-C*03:04	HARALPIQL	0.131	199.22	SB
chrX	47041409	C	A	RBM10:NM_005676	HLA-C*07:02	YYYDPKTGL	0.005	60.5	SB
chrX	47041409	C	A	RBM10:NM_005676	HLA-C*07:02	YYDPKTGLY	0.007	75.81	SB
chrX	47041409	C	A	RBM10:NM_005676	HLA-C*07:02	YYYDPKTGLY	0.041	86.94	SB
chrX	47041409	C	A	RBM10:NM_005676	HLA-C*07:02	YYDPKTGLYY	0.029	134.3	SB
